# Neuroprotection via Reduction in Stress: Altered Menstrual Patterns as a Marker for Stress and Implications for Long-Term Neurologic Health in Women

**DOI:** 10.3390/ijms17122147

**Published:** 2016-12-20

**Authors:** David Prokai, Sarah L. Berga

**Affiliations:** Department of Obstetrics and Gynecology, Wake Forest School of Medicine, Winston-Salem, NC 27157, USA; dprokai@wakehealth.edu

**Keywords:** stress reduction, functional hypothalamic amenorrhea, neuroprotection

## Abstract

Individuals under chronic psychological stress can be difficult to identify clinically. There is often no outwardly visible phenotype. Chronic stress of sufficient magnitude not only impacts reproductive function, but also concomitantly elicits a constellation of neuroendocrine changes that may accelerate aging in general and brain aging in particular. Functional hypothalamic amenorrhea, a phenotypically recognizable form of stress, is due to stress-induced suppression of endogenous gonadotropin-releasing hormone secretion. Reversal of functional hypothalamic amenorrhea includes restoration of ovulatory ovarian function and fertility and amelioration of hypercortisolism and hypothyroidism. Taken together, recovery from functional hypothalamic amenorrhea putatively offers neuroprotection and ameliorates stress-induced premature brain aging and possibly syndromic Alzheimer’s disease. Amenorrhea may be viewed as a sentinel indicator of stress. Hypothalamic hypogonadism is less clinically evident in men and the diagnosis is difficult to establish. Whether there are other sex differences in the impact of stress on brain aging remains to be better investigated, but it is likely that both low estradiol from stress-induced anovulation and low testosterone from stress-induced hypogonadism compromise brain health.

## 1. Introduction

Stress has many adverse health effects [1]. Unfortunately, individuals under chronic stress are often difficult to clinically identify, as there are few objective measures to define those that are chronically stressed. In women, phenotypic markers of chronic stress include menstrual irregularities, amenorrhea, and/or infertility due to hypothalamic hypogonadism. The endocrine signature of stress may be more likely to be reported as menstrual cycle changes rather than as complaints of stress per se. Herein, we focus on the pathobiological mechanisms mediating the link between chronic stress and reproductive compromise and show how stress-induced alterations in neuroendocrine secretory patterns may impact brain health and accelerate the onset of aging syndromes such as osteoporosis, vaginal atrophy, and dementia. Functional hypothalamic amenorrhea (FHA) is the most clinically evident example of the psychoneuroendocrinological condition, often termed functional hypothalamic hypogonadism. Our research has not only revealed the mechanisms mediating the link between chronic stress and FHA but has also established that stress reduction results in restoration of ovulation and amelioration of other neuroendocrine concomitants [2]. FHA is caused by chronic stress. While the stressors are often viewed as either metabolic or psychological, from a neuroendocrine perspective, this is a false dichotomy [2]. Regardless of the category, the final common pathway is activation of the limbic–hypothalamic–pituitary–adrenal axis [3] that then reduces central gonadotropin-releasing hormone (GnRH) drive [4,5]. The term “functional” is used to distinguish organic from behavioral causes and implies that, once the salient stressors are identified and their impact reduced, cortisol levels will normalize, GnRH drive will increase, and ovulatory ovarian function will resume [6]. FHA may well be the most common cause of amenorrhea in reproductive-age women [7].

## 2. Role of Stress in Functional Hypothalamic Amenorrhea (FHA)

There is evidence that stress is the proximate cause of FHA [6]. The combination of metabolic and psychological stressors causes a multitude of physiologic changes; the initiating signal that elicits and maintains the other neuroendocrine concomitants is a subtle, mostly nocturnal, increase in circulating cortisol [8]. Women with FHA have higher cortisol levels relative to eumenorrheic women and women with other forms of ovulatory dysfunction [6,9]. Notably, this hypercortisolism is also observed in the cerebrospinal fluid (CSF) of women with FHA [10]. Not only is the proportional rise greater in the CSF than in the circulation, the cortisol in the CSF is also unbound and thus more bioavailable. Therefore, the brunt of activated stress signaling may impact the brain more than peripheral tissues. Chronic hypoestrogenism from anovulation also compromises neural health and prevents age-appropriate bone accretion. Hypogonadism is due to stress-induced reduction in GnRH input leading to decreasing levels of luteinizing hormone (LH) and follicle-stimulating hormone (FSH) [2,6,11] to the extent that folliculogenesis is not supported and anovulation and secondary amenorrhea occur [2,6]. However, the suppression of GnRH occurs on a spectrum and lesser or intermittent “bouts” of reduced GnRH drive may manifest as luteal insufficiency and/or anovulatory cycles [12]. Therefore, the patient’s presenting symptoms to clinicians may be oligomenorrhea or infertility rather than amenorrhea [4,5]. Clinicians need to recognize that FHA is a diagnosis of exclusion and establishing the diagnosis requires a thorough evaluation of the patient.

Of note, stress and its resulting hormonal changes could trigger either undernutrition or overnutrition, depending on fuel availability, attitudes about food, and dietary behaviors such as bingeing, purging, overeating, or restricting. While the classic description of FHA is a thin woman who undereats and overexercises as a means of stress management, the new age profile may well be a high-achieving individual with overeating behaviors. Thus, chronic stress leads to a range of problematic eating patterns that could eventuate in either thinness or obesity. Obesity alone can suppress GnRH drive [13]. It is important to realize that not all anovulatory women who are obese have polycystic ovary syndrome and that some, possibly the majority, have “heavy” as opposed to “thin” FHA. From a systemic perspective, stress and obesity can act synergistically to increase inflammation [14]. This increased systemic inflammation over time has been associated with increased brain aging and an increased propensity towards neurodegeneration, such as syndromic Alzheimer’s disease (AD) [15].

## 3. Glucocorticoid Effects upon Reproductive Tissues

The health impact of chronic hypothalamic hypercortisolism and its concomitants remain to be fully characterized; however, it is clear that the impact extends beyond perturbations in menstrual cyclicity [16]. Of particular interest to health care providers for women is that stress and increased glucocorticoids have been shown to have profound effects on pregnancy and fetal development [17]. An association between maternal socioeconomic status, stress levels, and increased risks of preterm labor has also been described [18]. Stress increases expression of corticotrophin-releasing hormone (CRH), which in turn stimulates myometrial contractions via cytokine release [19,20,21], thereby potentiating labor [22,23,24]. Additionally, elevated maternal serum levels of CRH are seen in the second trimester of pregnancy in women who eventually deliver prematurely [25,26]. Stress reduction (e.g., group pregnancy care) has been shown to reduce rates of preterm labor [27,28,29,30]. In essence, both the magnitude and type of stressor elicit a constellation of neurohormonal alterations and accelerate molecular aging via regulation of telomerase [31].

Exogenous glucocorticoids (betamethasone and dexamethasone) are given for pregnancies at risk of preterm delivery, as these steroids accelerate fetal lung surfactant expression and release, thereby decreasing respiratory compromise due to atelectasis encountered by the premature newborns [32,33]. While synthetic glucocorticoids provide immediate fetal/neonatal benefit, repeated exposure not only negatively impact fetal outcomes, but emerging data suggest that glucocorticoid exposure in utero carries risks for diseases that will manifest in adulthood. Specifically, epidemiologic studies have shown that those exposed to excess glucocorticoids in utero display long-term health consequences due to accelerated aging [34,35,36,37]. Animal models have also shown persistent changes at the level of the glomeruli following betamethasone exposure, further demonstrating that the long-lasting effect of antenatal steroid exposure [38,39,40] is at least in part mediated via epigenetic mechanisms and consequences of accelerated aging at critical stages of fetal development [41].

## 4. Neuroprotection via Reduction in Stress

Stress management falls into two main categories: pharmacologic and nonpharmacologic, which can be employed concomitantly. Nonpharmacologic approaches such as cognitive behavioral therapy (CBT) should be considered as first line therapy because most behaviors that are stressful to self and others are initiated in response to cognitions [42]. CBT improves coping mechanisms and has been shown to restore ovarian function, including ovulation, in women who were previously amenorrheic due to reduced central GnRH drive [43]. The goal of CBT is to reduce allostatic load [44] by recognizing the stressful attitudes and behavior that cause excess activation of the adrenal axis and secondarily other alterations in neuroendocrine function. We have previously shown that CBT-induced restoration of ovarian function was accompanied by reductions in cortisol, particularly during the nocturnal phase, and other metabolic variables, including increased leptin and thyroid-stimulating hormone (TSH) independent of weight gain [45]. In this study, sixteen women with FHA were randomized to CBT or observation for twenty weeks [45,46]. Of the eight women treated with CBT, six resumed ovulating, one had partial recovery of ovarian function without evidence of ovulation, and only one did not display return of ovarian function. Two of the eight individuals not treated with CBT showed only partial return of ovarian function. In summary, stress causes more than an isolated disruption of reproduction function and stress management restores the panoply of neuroendocrine patterns. To the extent that chronic stress impairs brain function, then stress reduction will afford neuroprotection. Improvements in stress reduction and coping mechanisms may have both acute and chronic benefits to the individual and her offspring.

## 5. Molecular Consequences of Stress

An insidious consequence of the elevated cortisol that accompanies chronic stress is that the cellular machinery that estrogen would otherwise access is hijacked by the elevated glucocorticoids [47]. Using molecular expression, Bolt et al. showed that cortisol and estradiol utilized the same co-activators to “fine-tune” transcriptional responses [48].

Molecular analysis has shown that glucocorticoid and estrogen receptors have binding sites within the promotor elements of similar genes [49], and that glucocorticoid and estrogen receptor binding sites antagonistically regulate the promoters of many genes. Pathway analysis has shown significant overlap between the networks controlled by glucocorticoids and estrogen [50]. In addition to direct transcriptional regulation, antagonism of estradiol signaling by glucocorticoid may occur through regulation of chaperone proteins [51]. These molecular findings speak to the importance of lowering stress and cortisol. Therefore, not only do patients with FHA have lower levels of estrogen, but exogenous estrogen replacement may not reverse consequences of hypoestrogenism due to the antagonism of estrogen action by elevated glucocorticoids.

Hypercortisolism induces a state termed catabolism and women with FHA are catabolic rather than anabolic [52]. Catabolism is conferred and signaled by reduced thyroid hormone and growth hormone secretion [6]. The elevated cortisol that is central to FHA directly reduces TSH, thyroxine (T4), and thyronine (T3) levels. Cortisol suppresses the TSH response to thyrotropin-releasing hormone (TRH) [53].

A central theme is that chronic stress accelerates aging and, once an organism’s energy balance shifts toward survival, cellular upkeep suffers through switching from an anabolic to a catabolic state with suppression of the hypothalamic–pituitary–thyroidal (HPT) axis [52]. This metabolic reprioritization leads to diminished cellular repair and upkeep, which may contribute to the development of neurodegeneration and associated dementia. In Table 1, we summarize the metabolic patterns contributing to diminished brain health in women with FHA.

## 6. Stress and Neurodegenerative Diseases

Stress enhances sympathetic activity and activates the Hypothalamus–pituitary–adrenal (HPA) axis [54]. While such activation may foster acute survival, frequent and prolonged stimulation can have many deleterious consequences. The work of McEwen has focused on the “double edged” nature of stress in relation to the organism. When exposed to an acute stress, stress hormones released by the body seek to restore homeostasis in the face of a challenge. This is referred to as “allostasis” [55]. If the stress response is too frequent or the stress hormone response system is operating in an inefficient manner, there is a cost that must be paid by the organism. The price for maintaining equilibrium is referred to as the “allostatic load” [44,55]. We surmise that, in women with FHA, the systems that protect and foster neurologic health suffer as a consequence of chronic stress.

The resultant inflammation owing to chronic stress extends far beyond the reproductive axis and most certainly extends to the central nervous system (CNS) [7]. Microglia play a critical role [56]. As the principal immune cells of the CNS, they are vital to protecting the brain against stressful stimuli. Microglia are rich in steroid hormone receptors and estrogen has been shown to be a potent regulator of microglial activity [56,57]. Estrogen has been shown to limit the pro-inflammatory state of microglia in response to bacteria, viruses, and hypoxia [58,59,60]. Women with FHA, with hypoestrogenism, hypothyroidism, and hypercortisolism, are at risk for being in a pro-inflammatory state. If so, the brains of women with FHA may be more susceptible to routine physiologic or psychological stressors. Williams et al. have shown that stressors interact synergistically to amplify other stressors [61].

Unchecked neuroinflammation has been identified as one of the primary causes for impaired neurogenesis and the loss of neuronal stem cells [62]. In addition to impairing neurogenesis, persistent inflammation impairs neuronal stem cell survival and differentiation and promotes their age-related decline and neurodegenerative diseases [63,64,65].

For example, neuroinflammation is known to be involved in the pathology of AD and to accelerate the processing of amyloid precursor protein into neurotoxic amyloid-β peptides and their relocalization/deposition to plaques and to promote tau-protein hyperphosphorylation [66]. Neuroinflammation is also thought to be one of the central mechanisms that predisposes towards AD [67]. By restoring glucocorticoid exposure to physiological patterns, stress reduction may foster better acute and chronic neural health [68]. Thus, managing stress has benefits beyond fertility and may reduce brain aging.

## 7. Conclusions

While direct evidence may be lacking, FHA is likely to compromise brain health via several mechanisms. In particular, estradiol plays a critical role in maintaining brain architecture and metabolism, and chronically low levels associated with anovulation may impair brain health. The high systemic and CSF levels of cortisol due to stress likely cause direct neuronal and glial toxicity and potentiate early apoptosis in vulnerable regions of the brain. Alterations in the thyroid axis of women with FHA likely impair neurogenesis and synaptic connectivity. Finally, prolonged stress and its concomitant hormonal effect produce a catabolic state that may render the individual more susceptible on subsequent stressors including infection.

The development of amenorrhea and oligoamenorrhea afford the clinician an early warning sign and allow identification of patients who would benefit from interventions to reduce the burden of stress and treat FHA. Decreases in stress are predicted to not only restore fertility but also to reduce neuroinflammation and protect against premature brain aging and possibly even against neurodegenerative diseases. With a reduction of psychological stress, and a concomitant reduction in glucocorticoids, one can go beyond neuroprotection and eventually achieve “neuroprevention”.

## Figures and Tables

**Table 1:** Important metabolic aberrations in Functional Hypothalamic Amenorrhea (FHA) that may contribute to compromised brain health.

Metabolic Aberrations in FHA that May Contribute to Long-Term Neurodegeneration
Elevated cortisol [9,10]
Hypoestrogenism [2]
Catabolism [51]
Hypothalamic hypothyroidism [6]

## Abbreviations

AD	Alzheimer’s disease
CBT	Cognitive behavioral therapy
CNS	Central nervous system
CRH	Corticotrophin-releasing hormone
CSF	Cerebrospinal fluid
FHA	Functional hypothalamic amenorrhea
GnRH	Gonadotropin-releasing hormone
HPA	Hypothalamus–pituitary–adrenal
TSH	Thyroid-stimulating hormone
